# Disruption in functional networks mediated tau spreading in Alzheimer’s disease

**DOI:** 10.1093/braincomms/fcae198

**Published:** 2024-06-13

**Authors:** Fardin Nabizadeh

**Affiliations:** School of Medicine, Iran University of Medical Sciences, Tehran 441265421414, Iran

**Keywords:** Alzheimer’s disease, tau, fMRI, connectivity

## Abstract

Alzheimer’s disease may be conceptualized as a ‘disconnection syndrome’, characterized by the breakdown of neural connectivity within the brain as a result of amyloid-beta plaques, tau neurofibrillary tangles and other factors leading to progressive degeneration and shrinkage of neurons, along with synaptic dysfunction. It has been suggested that misfolded tau proteins spread through functional connections (known as ‘prion-like’ properties of tau). However, the local effect of tau spreading on the synaptic function and communication between regions is not well understood. I aimed to investigate how the spreading of tau aggregates through connections can locally influence functional connectivity. In total, the imaging data of 211 participants including 117 amyloid-beta-negative non-demented and 94 amyloid-beta-positive non-demented participants were recruited from the Alzheimer’s Disease Neuroimaging Initiative. Furthermore, normative resting-state functional MRI connectomes were used to model tau spreading through functional connections, and functional MRI of the included participants was used to determine the effect of tau spreading on functional connectivity. I found that lower functional connectivity to tau epicentres is associated with tau spreading through functional connections in both amyloid-beta-negative and amyloid-beta-positive participants. Also, amyloid-beta-PET in tau epicentres mediated the association of tau spreading and functional connectivity to epicentres suggesting a partial mediating effect of amyloid-beta deposition in tau epicentres on the local effect of tau spreading on functional connectivity. My findings provide strong support for the notion that tau spreading through connection is locally associated with disrupted functional connectivity between tau epicentre and non-epicentre regions independent of amyloid-beta pathology. Also, I defined several groups based on the relationship between tau spreading and functional disconnection, which provides quantitative assessment to investigate susceptibility or resilience to functional disconnection related to tau spreading. I showed that amyloid-beta, other copathologies and the apolipoprotein E epsilon 4 allele can be a leading factor towards vulnerability to tau relative functional disconnection.

## Teaser

Nabizadeh aimed to investigate how the spreading of tau aggregates through connections can locally influence functional connectivity. The findings provide strong support for the notion that tau spreading through connection is locally associated with disrupted functional connectivity between tau epicentre and non-epicentre regions independent of amyloid-beta pathology.

## Graphical Abstract

**Graphical Abstract:**
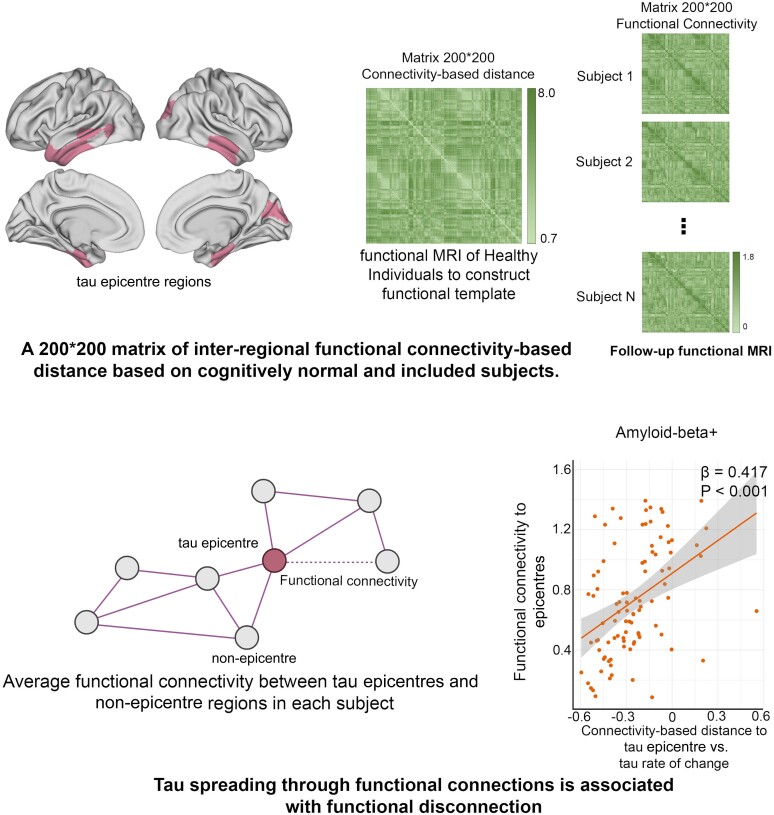

## Introduction

Alzheimer’s disease is the most common neurodegenerative disease, which is slowly progressive with variable clinical presentation.^1^ The emergence of Aβ plaques sets off a chain of events including the deposition of neocortical tau pathology with ensuing synaptic dysfunction and neuronal disconnection.^2,3^ In recent years, the concept of Aβ plaque deposition as the pioneering event in Alzheimer’s disease has been challenged as it is believed that tau pathology might precede Aβ plaque deposition at a subthreshold biomarker detection level in time, followed by independent Aβ plaque deposition rising above the biomarker detection threshold and accelerating tauopathy.^3^ However, the interaction and sequence of these pathophysiological features on functional brain networks during the predementia period are not well understood. Since Alzheimer’s disease is considered disconnection syndrome, it is crucial to understand the interrelation between key pathological aggregates of Alzheimer’s disease, especially tau tangles and functional brain connectivity.^4,5^

Post-mortem studies suggest that tau pathology is commonly present in the medial temporal lobe (MTL) and probably first confined in the entorhinal cortex (EC) and locus coeruleus and then propagating to the hippocampus and beyond to limbic and associated cortices.^6^ The sequential spread of tau aggregates across anatomically related regions fostered the idea that misfolded tau released from neurons and taken up by connected neurons thereby triggering a self-propagating tau spreading cascade (known as ‘prion-like’ properties of tau), which was later confirmed by multiple experimental studies.^7^ It was demonstrated that injection of human tau into the brains of β-amyloid-expressing transgenic rodents leads to the accumulation of tau in brain regions that were anatomically connected to the injection site.^4,8^ In support, studies combining PET and functional MRI (fMRI) data demonstrated that future tau spreading is strongly related to functional connectivity (FC).^9,10^ Together, transsynaptic tau pathology spread through connection between actively communicated regions provides an opportunity to map the spatial distribution and follow and tackle the initial progress of tau aggregation.

Efficient information transfer along the neuronal groups within functionally specialized brain areas results in normal cognitive function.^11,12^ Disruption of this information transfer leads to cognitive dysfunction in multiple neurological disorders including Alzheimer’s disease.^13,14^ The Aβ plaques and tau neurofibrillary tangles result in the progressive degeneration and shrinkage of neurons, along with synaptic dysfunction in distinct cortical and subcortical brain regions. These findings indicate that Alzheimer’s disease may be conceptualized as a ‘disconnection syndrome’, characterized by the breakdown of neural connectivity within the brain.^15,16^ Despite the consensus of the previous findings regarding the brain functional network breakdown due to the pathology, the exact underlying process is little known. Some initial investigations revealed that the tau deposition spatial pattern overlaps with functional networks such as default mode network (DMN) and frontoparietal network (FPN) while it was unclear whether tau accumulation differentially affects this network or not.^17,18^ Since then the effect of tau pathology on FC comes into sharp focus. A study by Cope *et al*.^19^ demonstrated that an increase in tau-PET is associated with reduced connectivity within multiple networks and between highly interconnected hubs. Another study involving cognitively normal (CN) old adults revealed a positive relationship between increased posterior DMN connectivity to non-DMN brain regions with elevated global amyloid levels, as well as localized tau levels.^20^ Overall, it can be drawn from these findings that tau spreads through functional networks in the early stages resulting in faltered connectivity within the networks. However, there is no evidence of the local effect of tau spreading on the functional connections mediated by tau accumulation. It is critical to elucidate the effect of tau spreading on the synaptic function and communication between regions.

To address the knowledge gap, I leveraged the cross-sectional and longitudinal tau-PET, resting-state fMRI connectomics from the Alzheimer’s Disease Neuroimaging Initiative (ADNI) data set. First, I tested how the spreading of tau aggregates through connections can locally influence FC. Second, I clustered participants based on whether they were resilient or susceptible to functional disconnection due to the tau spreading. Considering that Alzheimer’s disease autopsy studies revealed widespread non-Alzheimer’s disease copathology,^21^ I hypothesized that resilience or susceptibility to functional disconnection due to tau spreading might be attributable to a spectrum of mixed disease burdens such as TDP-43 and vascular diseases or even genetic vulnerability. Overall, my study suggests that tau spreading through functional networks can locally cause disconnection within connected regions. Also, I found that resilience or susceptibility to functional disconnection due to the tau spreading can be driven by APOE ε4 allele, microglia pathology, TDP-43 and vascular disease. My study provides a deep insight into how tau pathology contributes to disrupted brain connectivity in the early stages of Alzheimer’s disease.

## Materials and methods

### Participants

The ADNI is a collaborative research project that began in 2003 as a partnership between public and private organizations. Its main goal has been to explore the possibility of using various medical imaging techniques, along with other biological markers and clinical assessments, to measure the progression of mild cognitive impairment (MCI) and early-stage Alzheimer’s disease. For the latest information, please visit www.adniinfo.org. The current study made use of data from ADNI, which received approval from all relevant ethics boards associated with the initiative (refer to Reporting Summary). All participants provided informed consent and were compensated for their participation. The study focused on individuals with longitudinal tau-PET, Aβ-PET (florbetaben or florbetapir) and CSF p-tau181 data available in the ADNI database as of July 2021. This included 219 participants out of a total of 777 individuals with longitudinal tau-PET and resting-state fMRI. Among these, only eight were diagnosed with Alzheimer’s disease dementia and, due to the small sample size, they were excluded from the study. The final sample of 211 participants consisted of both cognitively unimpaired (CU) and MCI participants. ADNI assessed the participants’ clinical status: CU participants had an Mini-Mental State Evaluation (MMSE) score above 24, a Clinical Dementia Rating (CDR) score of 0 and no signs of depression, while participants with MCI had an MMSE score above 24, a CDR of 0.5, objective memory impairment based on education-adjusted Wechsler Memory Scale II and preserved activities of daily living. Since the data in this paper were obtained from the ADNI database (adni.loni.usc.edu), it does not include any research involving human or animal subjects.

### Image acquisition and processing

The procedures for acquiring PET and MRI images in the ADNI cohort are documented in detail elsewhere (http://adni.loni.usc.edu/methods/documents/). All image processing was performed locally, involving realignment, averaging, reslicing to 1.5 mm^3^ and smoothing to a resolution of 8 mm^3^ full width at half maximum. The PET images were spatially aligned to the closest structural T_1_-weighted MRI scans. Standardized uptake value ratio (SUVR) PET images were generated, using the inferior cerebellum as the reference region for the flortaucipir tracer and the whole cerebellum for the florbetapir and florbetaben tracers. Furthermore, each participant’s T_1_-weighted MRI image was registered to the standardized Montreal Neurological Institute (MNI) template space using the ANTs software version 2.3.1. Applying these same registration parameters, the SUVR PET images were also transformed into the common MNI space to facilitate subsequent analyses. This processing pipeline allowed for the spatial normalization of both the structural MRI and functional PET data, enabling region-of-interest analyses and group-level comparisons within a standardized anatomical framework.

The study established Aβ positivity thresholds based on the reference standards defined by the ADNI PET score. Specifically, a global cortical SUVR value of 1.11 was used as the cut-off for the florbetapir tracer, while a SUVR of 1.08 was the threshold for the florbetaben tracer, both referenced to the whole cerebellum.^22^ In order to perform the analysis of all participants together, despite the use of two different Aβ PET tracers, I converted the global Aβ SUVR values to the Centiloid scale.^23^

### Regional measures and rate of change of PET

The tau-PET images were parcellated into Schaefer functional atlas, openly available on nilearn (nilearn.datasets.fetch_atlas_schaefer_2018).^24^ The average SUVR values were measured for these 200 parcels, following the masking of each region with a grey matter mask to reduce the effect of binding from the white matter or CSF on the final values used in the analyses.

I measured the tau-PET SUVR and tau rate of change according to the method described in Pichet Binette *et al.*^25^ study. Participants underwent two to four tau-PET scans (see Table 1).

**Table 1:** Demographic and clinical characteristics

Variable	Aβ-negative non-demented (*n* = 117)	Aβ-positive non-demented (*n* = 94)
Age (years)	68.8 ± 6.2	71.4 ± 6.3
Female (%)	71 (60%)	49 (52%)
Education (years)	16.3 ± 2.5	16.4 ± 2.6
APOE ε4 carriers (%)	30 (26%)	55 (67%)
MMSE	28.5 ± 1.2	28.4 ± 1.6
Tau-PET follow-up time (years)	3.3 ± 1.6	2.6 ± 1.2
MCI (%)	74 (63%)	74 (79%)

Data are presented as mean ± SD unless specified otherwise. APOE ε4, apolipoprotein E genotype (carrying at least one ε4 allele); MMSE, Mini-Mental State Evaluation; MCI, mild cognitive impairment.

### Identifying tau-PET epicentres

Tau-PET epicentres were identified via using Gaussian mixture models (GMMs) on the baseline tau-PET data. The full method was described in Pichet Binette *et al.*^25^ study. The probability of falling on the right-most distribution (‘high’ tau-PET distribution) was extracted for each participant.^9,10,26^ As this right-most distribution likely reflects an abnormal tau-PET signal, the GMM probability serves as a probabilistic measure of tau positivity, obviating the need for *a priori* thresholds. The GMM probabilistic value was then multiplied with the tau-PET SUVR to obtain a cleaned SUVR score devoid of non-specific signals. To identify individual-level tau-PET epicentres, the regions with the highest SUVR were selected, weighted by GMM. Specifically, the 10 regions exhibiting the greatest probability-weighted SUVR values were chosen and subsequently utilized for further connectivity-based analyses.^26^

### FC analyses

In order to explore the potential association between tau-PET accumulation and functional brain architecture, I constructed a normal template FC matrix using data from 69 CU individuals in the ADNI cohort who were Aβ-negative and exhibited low tau-PET binding (global SUVR < 1.3).^27^ Also, a template matrix was constructed for each included subject using follow-up fMRI data (non-baseline) to investigate FC between pairs of regions. The methodology for deriving the template matrix has been detailed elsewhere. In brief, the process involved realigning functional images to the first volume and coregistering them to the native T_1_ images. Further processing steps included detrending, band-pass filtering (0.01–0.08 Hz) and regressing out nuisance covariates (average white matter and CSF signal, as well as motion parameters). Frames with frame displacement >1 mm (along with the frame prior and the two subsequent frames) were scrubbed, and only participants with <30% of censored data were retained. FC matrices were constructed based on the functional Schaefer atlas of 200 parcels. Fisher-*Z* correlations between time–series averaged across voxels within each region of interest (ROI) were computed to assess subject-specific FC matrices. These individual matrices were then averaged and thresholded at 30% density. Subsequently, for the construction of a normal connectivity matrix of 69 CU participants, the average FC was transformed into a distance-based connectivity matrix, where shorter path lengths between ROIs indicated stronger connectivity.^28^

To establish a link between normal FC and tau aggregate accumulation, I calculated the distance-based FC of brain regions to the tau epicentres as defined previously. At both the group and individual levels, I correlated the rate of tau-PET change in each region with its connectivity-based distance to the tau epicentres across all remaining brain regions that were not epicentres (*n* = 190). I measured the association between tau-PET accumulation and connectivity to tau epicentres across, as a measure of connectivity-mediated tau spreading (standardized *β*). Negative *β*-values were anticipated, signifying that stronger connectivity (represented as smaller values due to the conversion of connectivity measures to distance-based) would be associated with greater tau-PET change. It is noteworthy that repeating this same approach while adjusting for Euclidean distance between ROIs, or when using a normal FC matrix based on partial correlations as a template, yielded consistent results. Moreover, to investigate the link between standardized *β*-value and FC in each participant, I only used FC between all tau non-epicentre regions (*n* = 190) to epicentres.

### Statistical analysis

The analyses were conducted separately in the Aβ-positive, Aβ-negative and all individuals. All analyses were conducted using R version 4.0.5, with the main packages including stats v4.0.5, lme4 v1.1-30 for linear mixed effect models, mediation v4.5.0 for mediation analyses and ggplot2 v3.3.6 for creating plots. Brain renderings were generated using the Connectome WorkBench software v.1.5.0 and BrainNet Viewer.^29^

In the initial phase, I conducted an investigation into the primary factors associated with the FC to epicentres. My focus was on the baseline Aβ-PET, tau-PET and tau-PET rate of change. I used linear regression models, with FC to epicentres as the dependent variable and age, sex, education and APOE ε4 allele as covariates.

For each participant, linear models were fitted across all non-epicentre regions to assess the association between connectivity to tau epicentres and tau-PET rate of change, resulting in a *β*-value. Then, I measured the association between this *β*-value and FC to epicentre while adjusting for age, sex, education and APOE ε4 allele (carrier versus non-carrier) as covariates. Based on the outcomes from the linear models, I also assessed the mediating effect of Aβ-PET on the association between *β*-value and FC to epicentres. Mediation analyses were conducted using the mediation package (version 4.5.0) in R. All paths of the mediation model were controlled for the effects of age and sex. The significance of the mediation effect was determined through 1000 bootstrapping iterations.

The spatial distributions between *β*-value and FC to epicentre relationship mismatch were examined through the clustering of residuals from a regression model of *β*-value versus FC to epicentre. Robust linear regressions of individual *β*-value versus FC to epicentres were conducted in each of seven brain networks (visual, somatomotor, dorsal attention, ventral attention, limbic, FPN and DMN) to produce *β*-value versus FC to epicentre mismatch residuals (in units of FC to epicentre). A bi-square weighting function was employed to minimize the influence of outliers in the robust regression. To mitigate the potential influence of outliers on the clustering analysis, I implemented a discretization procedure for the regression residuals. For each ROI and participant, the residual values were categorized based on a predefined threshold. Specifically, if the residual exceeded 0.7 SD from the regression line, either in the positive or negative direction, it was assigned a value of −1 or 1, respectively. All other residuals that fell within the 0.7 SD cut-off were assigned a value of 0. This discretization process resulted in an array of seven network-level variables across the 211 participants, where each entry represented the discretized residual status for that particular network. By converting the residuals into a categorical format, I aimed to reduce the potential distorting effects that extreme outliers might have had on the subsequent clustering analysis, thereby enhancing the robustness of the identified patterns.

The discretized residuals were used as inputs for Ward’s agglomerative hierarchical clustering, implemented using the hclust and cluster packages in R (v4.0.5). The goal was to create groups based on the mismatch between *β*-values and FC to the defined epicentres.^30^ The optimal number of clusters was determined through elbow and silhouette analyses, both of which suggested that *k* = 4 clusters provided the best balance between within-cluster similarity and between-group variation in the specific regional patterns.^31^ Lower cluster solutions were not selected, as they would not have captured as much of the underlying between-group differences. Additionally, PCA was performed on the discretized residuals to achieve dimensionality reduction. This clustering and dimensionality reduction approach allowed me to identify distinct subgroups based on the discrepancies between the measured *β*-values and the FC to the identified tau-PET epicentres, potentially revealing important insights into the regional patterns of tau pathology and functional network alterations.

The ANOVA was utilized to compare variables across the clustered groups. Covariates included age, sex and education. Multiple test adjustment was carried out using Benjamini–Hochberg correction with a false discovery rate of 0.05 for pairwise comparisons between the clustered groups. Box plots were employed to display the data points as dots, with the median as the middle box line, the first quartile (Q1) and third quartiles (Q3) as box edges (indicating the interquartile range, IQR) and whiskers as the minimum–maximum points. Genotype frequency comparisons were performed using *χ*^2^ tests.

I used ADNI-MEM (available in ADNI) to test whether the clustered groups represent different baseline and longitudinal cognitive declines.^32^ The study investigated longitudinal changes in cognitive performance using linear mixed effects modelling. This statistical technique allowed for the inclusion of participant-specific random intercepts, which accounted for individual differences at the baseline assessment. The model incorporated several predictor variables, including the initial cognitive score measured at the time of neuroimaging, the elapsed time since the scan, membership in distinct clusters or subgroups and the interaction between cluster and time. Demographic factors such as sex, age and education level were also included as covariates.

## Results

### Study design and participants

**Figure 1:**
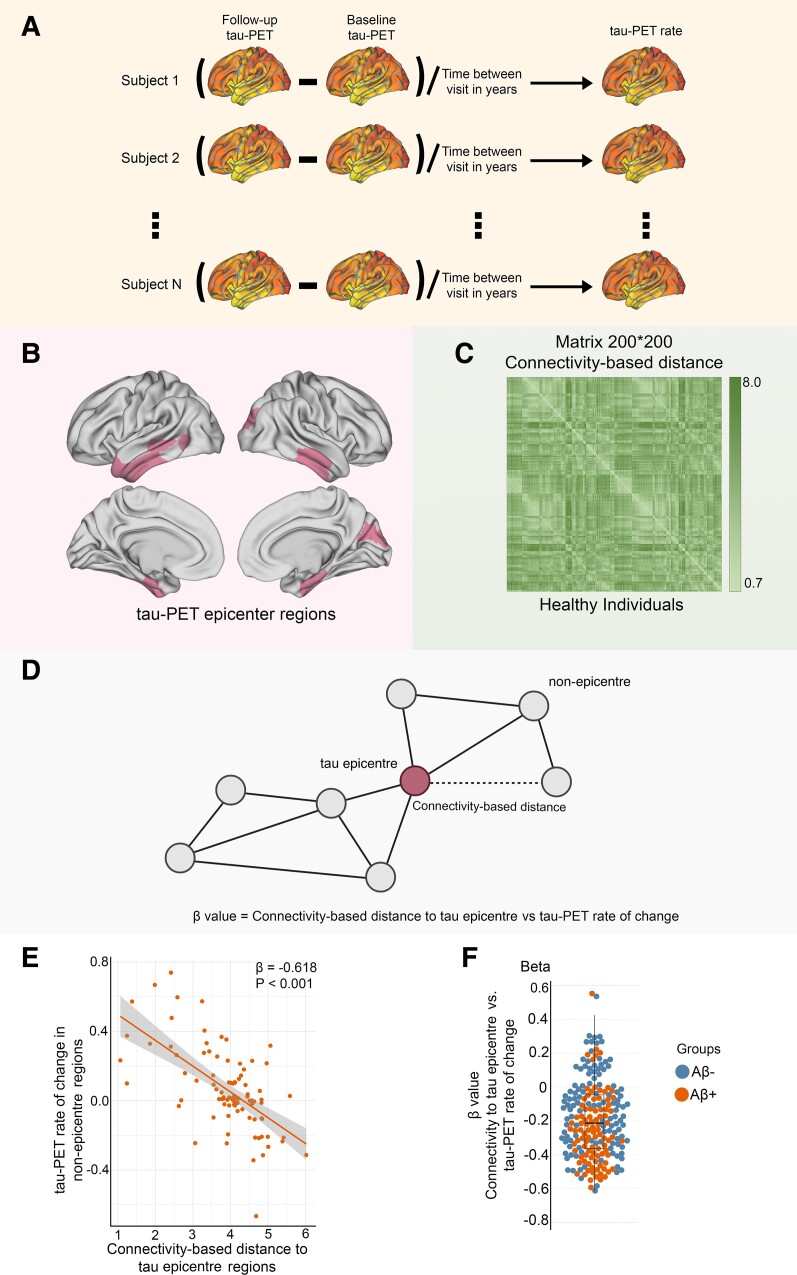
**Assessment of tau-PET epicentres and connectivity-based accumulation of tau aggregates.** (**A**) Assessment of tau-PET rate of change. Tau-PET rate of change was measured as the ROI-wise difference in baseline and follow-up tau-PET divided by the time interval between visits in years. (**B**) Representation of tau epicentres on glass brain in Aβ-positive participants. (**C**) A 200 × 200 matrix of interregional FC-based distance based on 69 CN subjects from the ADNI data set. The subject-specific connectivity matrices measured by Fisher-*Z* transformation on ROI-specific preprocessed fMRI time series were then averaged across individuals and subjected to a thresholding process, where only the strongest 30% of connections were retained. Finally, the matrices were transformed to connectivity-based distance, with shorter distances indicating higher levels of connectivity. (**D**) Hypothetical tau spreading through functional connections. In the model, tau aggregates spread from epicentres (regions with the highest level of tau aggregates in each subject) to non-epicentre regions through functional networks. The *β*-value from the correlation analysis between the rate of change in tau-PET and the connectivity-based distance to epicentres in each subject represents the tau spreading through interconnected regions. (**E**) The group-level analysis of the correlation between connectivity-based distance to the tau epicentres, as visualized on the glass brain in **B**, and the tau-PET rate of change across the entire brain in Aβ-positive participants (linear regression, *n* = 94). Each dot represents a distinct brain region. The results reveal that there is a higher rate of tau accumulation in regions that are functionally closer to the tau epicentres. (**F**) The individual level of the same analysis (**A** and **B**) is represented (linear regression, *n* = 211). The *β*-value from the correlation analysis between the rate of change in tau-PET and the connectivity-based distance to epicentres across all brain regions in each individual is displayed in the box plot (average: −0.208). Based on these results, tau spread across functional connections. A more negative *β*-value means a greater tau accumulation rate in the regions that are functionally closer to the tau epicentres. All linear regressions performed were two sided, without adjustment for multiple comparisons, and error bands correspond to the 95% confidence interval. The linear models adjusted for the effect of age, sex, education and APOE ε4 allele. Aβ, amyloid-beta.

I included a discovery sample consisting of 211 participants from the ADNI database. These participants had longitudinal AV1451 tau-PET and follow-up resting-state fMRI data (non-baseline visit) available. To ensure coverage of the Alzheimer’s disease continuum, which is defined by abnormal Aβ, our sample included 43 CN Aβ-positive individuals and 74 individuals with MCI who were Aβ-positive. Additionally, a total of 94 Aβ-negative participants including 20 CN and 74 MCI participants were included. All main analyses were conducted separately in the Aβ groups and all participants with all results in supplementary information. The PET data were segmented into 200 cortical ROIs using the Schaefer brain atlas.^24^ The accumulation of tau aggregates was quantified as the rate of change in tau-PET over time (SUVR/year) within each brain region, employing linear mixed effect models (Fig. 1A). The average follow-up time of tau-PET was 1.83 ± 1.23 years. Furthermore, the average time interval between baseline and follow-up visits of cross-sectional fMRI data was 1.94 ± 1.66.

### Connectivity-based accumulation of tau aggregates

Based on previous studies, functional architecture can be a significant modifier for tau accumulation.^9,25,33^ I, therefore, investigated whether the FC of the brain can be affected by connectivity-based accumulation of tau aggregates (tau spreading through functional networks). Initially, I identified participant-specific tau-PET epicentres, which were defined as the top 10 regions with the highest tau-PET SUVR probability as determined by Gaussian mixture modelling at baseline (Fig. 1B). The association between accumulation of tau aggregates in the remaining 190 regions and the strength of FC to these epicentres using functional connectome of healthy individuals was then assessed (Fig.1C and D). First, I applied this model at the group level and observed that regions that exhibited stronger connectivity to the epicentres, as indicated by shorter distance-based connectivity, had higher rates of accumulation of insoluble tau aggregates (Fig. 1E; Supplementary Fig. 1A).

In the next step, the model was performed at the individual level by defining tau epicentres for each participant. I found that regions with stronger connectivity (shorter distance-based connectivity) to the tau epicentres, as determined by baseline tau-PET had higher rates of tau aggregate accumulation (Fig. 1D). This association between connectivity to the epicentres and tau aggregate accumulation across all non-epicentre regions was supported by negative *β*-values (as displayed in Fig. 1F).

### Aβ and tau-PET are associated with FC to tau epicentres

**Figure 2:**
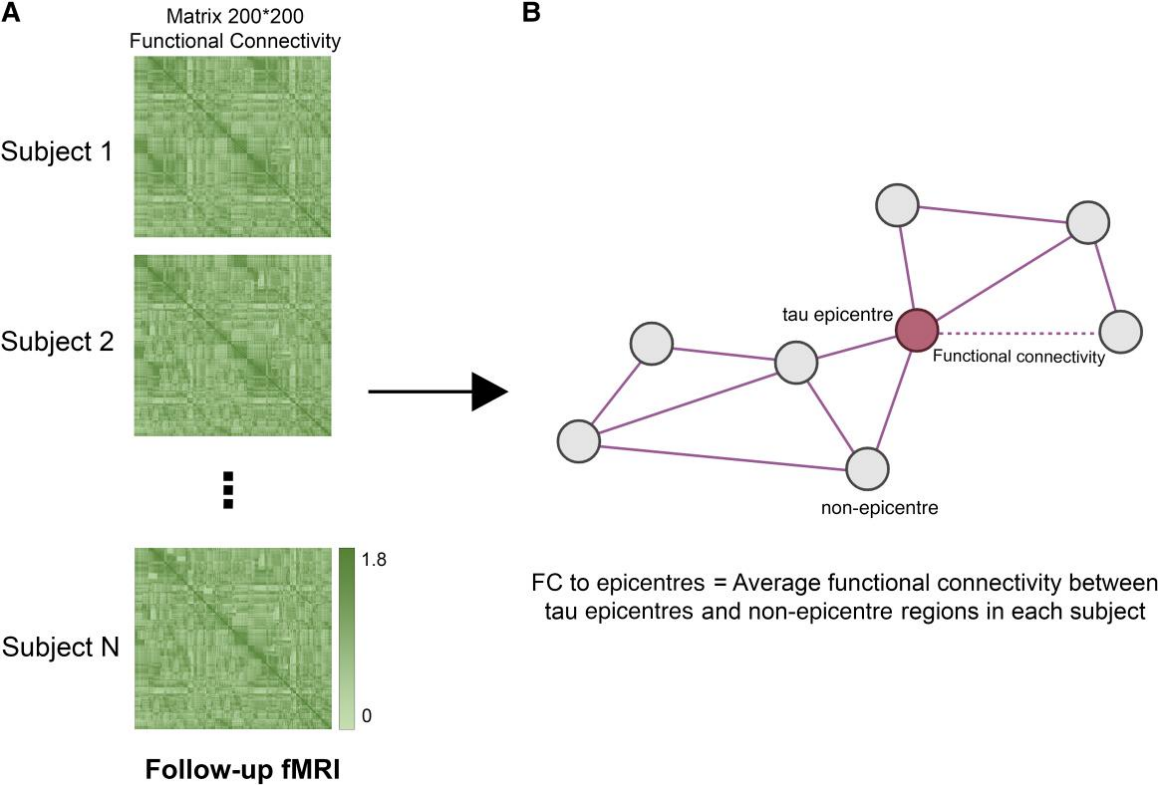
**FC to epicentres.** (**A**) A 200 × 200 matrix of interregional FC for each participant was measured using follow-up fMRI data (imaging data of baseline visits were excluded). (**B**) For each subject, the average FC of non-epicentre regions to tau-PET epicentre regions was measured (FC to epicentres). FC to epicentres can directly represent the FC of interregional connections that are pathways for the hypothetical tau spreading model (epicentre to non-epicentre regions). fMRI, functional MRI; FC, functional connectivity.

I tested whether Aβ or tau-PET is associated with subsequent FC between tau epicentre and non-epicentre regions. First, subject-specific FC metrics were obtained on preprocessed follow-up resting-state fMRI using the Schaefer brain atlas with 200 ROIs (Fig. 2A).^24^ Using the 200 × 200 matrix of interregional FC for each participant, I calculated FC to subject-specific tau epicentres (Fig. 1B) for each participant to only investigate the involved connections in tau spreading (Fig. 2B).

**Figure 3:**
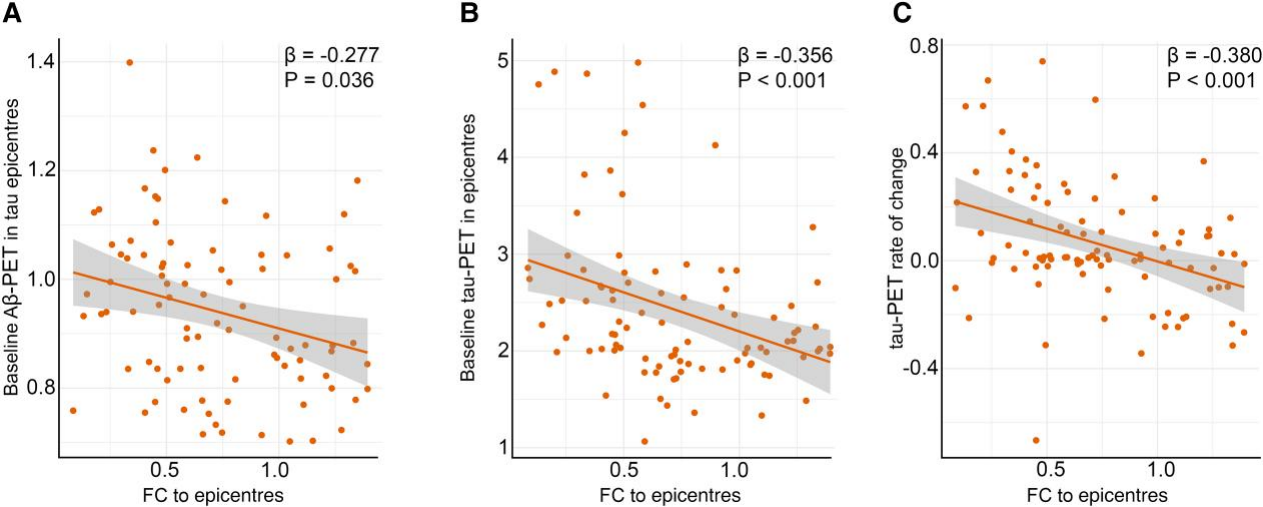
**FC to epicentres is associated with Alzheimer’s disease pathological hallmarks.** (**A**) Associations between baseline Aβ-PET in tau epicentre regions and FC to epicentres in Aβ-positive participants represented on the scatter plot (linear regression, *n* = 94). Each dot represents an individual. (**B**) Associations between baseline tau-PET in epicentre regions and FC to epicentres in Aβ-positive participants represented on the scatter plot (linear regression, *n* = 94). Each dot represents an individual. (**C**) Associations between baseline tau-PET rate of change and FC to epicentres in Aβ-positive participants represented on the scatter plot (linear regression, *n* = 94). Each dot represents an individual. These results (**A** and **B**) revealed that the Aβ and tau deposition in regions that have the highest level of tau aggregates is associated with lower average connectivity of non-epicentre to epicentre regions for tau-PET. Also, the observed negative association (**C**) between tau-PET rate of change and FC to epicentres suggests that faster overall tau accumulation is associated with altered FC to tau epicentre regions. All linear regressions performed were two sided, without adjustment for multiple comparisons, and error bands correspond to the 95% confidence interval. The linear models adjusted for the effect of age, sex, education and APOE ε4 allele. Aβ, amyloid-beta; fMRI, functional MRI; FC, functional connectivity.

Across Aβ-positive participants, baseline Aβ-PET in tau epicentres was negatively associated with FC to epicentres (Fig. 3A). However, global and non-epicentre Aβ was not associated with FC to epicentres in all analysed groups (Supplementary Fig. 1). These findings demonstrate that Aβ only in tau epicentres is associated with subsequent disruption in FC. Next, I investigated the possible association between tau-PET and FC to epicentres. Based on linear regression models, the level of tau-PET in epicentres and overall tau-PET rate of change were negatively associated with FC to epicentres in the Aβ groups (Fig. 3B and C; Supplementary Fig. 1C and E). The results of further analysis on the potential associations between FC to epicentres and Aβ- and tau-PET are provided in Supplementary Fig. 1.

### Tau spreading is associated with connectivity to tau epicentres

**Figure 4:**
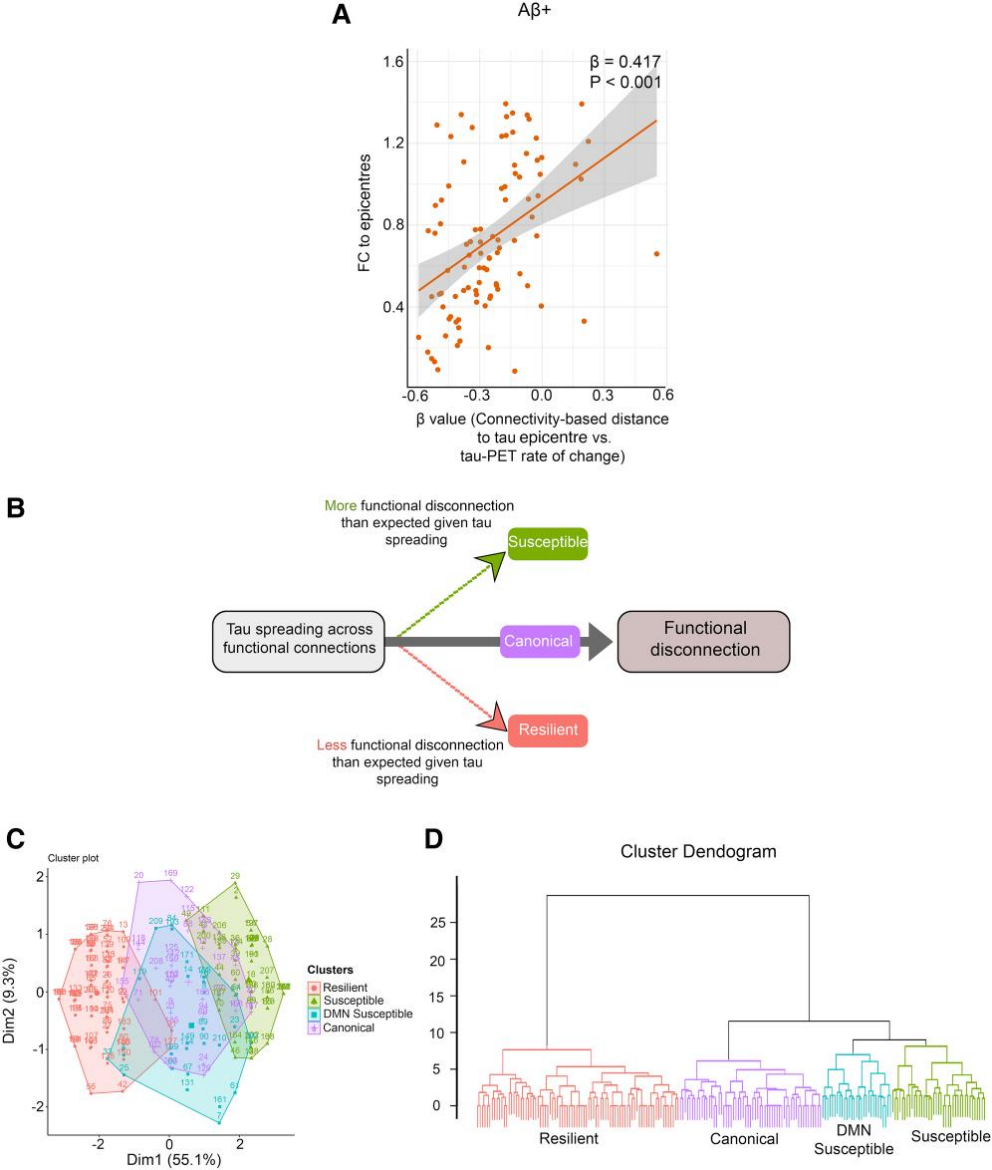
**Association between tau spreading and FC.** (**A**) Scatter plot of the associations between FC to epicentres and the *β*-values of epicentre connectivity to tau-PET rate of change in Aβ-positive participants (linear regression, *n* = 94). Each dot represents an individual. (**B**) Schematic of the proposed relationship between tau spreading and functional disconnection (FC between tau-PET epicentre and non-epicentre regions). Subjects with more functional disconnection given tau spreading considered susceptible. Also, subjects with lower functional disconnection given tau spreading considered resilient. (**C**) Consistent clustering by tau spreading and functional disconnection relationship of seven brain networks (visual, somatomotor, dorsal attention, ventral attention, limbic, FPN and DMN) and patient residuals is visually depicted by (**C**) PCA and (**D**) dendrogram. Aβ, amyloid-beta; fMRI, functional MRI; FC, functional connectivity.

I hypothesize that the spreading of tau aggregates through connections can locally influence FC. Thus, I investigated the potential association between connectivity-based accumulation of tau aggregates (*β*-value) and FC to epicentres to show the local effect of tau spreading on the involved connections. It was found that lower FC to epicentres is associated with a stronger association between connectivity to the epicentre and tau-PET rate of change in non-epicentre ROIs (*β*-value) in all analysed groups (Fig. 4A; Supplementary Fig. 1G).

Given that Aβ increases in early Alzheimer’s disease prior to the formation of insoluble tau aggregates in neocortical regions and affects FC,^34^ I tested whether Aβ measures mediate the association between tau spreading (*β*-value) and FC to epicentres. I found that Aβ-PET in tau epicentres mediated 11% of the association tau spreading (*β*-value) and FC to epicentres suggesting a partial mediating effect of Aβ deposition in tau epicentres on local effect of tau spreading on FC in Aβ-positive participants (Supplementary Fig. 2C). The result was not replicated in Aβ-negative participants (Supplementary Fig. 2G). Based on the analysis, there was no mediating effect for global and non-epicentre Aβ-PET on the association of tau spreading (*β*-value) and FC to epicentres (Supplementary Fig. 2).

### Tau spreading and functional disconnection relationship clustering

I measured the relationship between hypothetic tau spreading and FC to epicentres by regression analysis in each of the seven networks (visual, somatomotor, dorsal attention, ventral attention, limbic, FPN and DMN). Clustering on tau spreading and functional disconnection regression residuals resulted in four groups (Fig. 4B). The groups were labelled based on the pattern of metabolic resilience or vulnerability to functional disconnection given the relative tau spreading. A principal component analysis (PCA; Fig. 4C) and a dendrogram visualized the within-group similarity across clustered patients (Fig. 4D). Also, tau spreading and functional disconnection relations were seen in residual heatmaps across seven networks (Supplementary Fig. 3).

**Figure 5:**
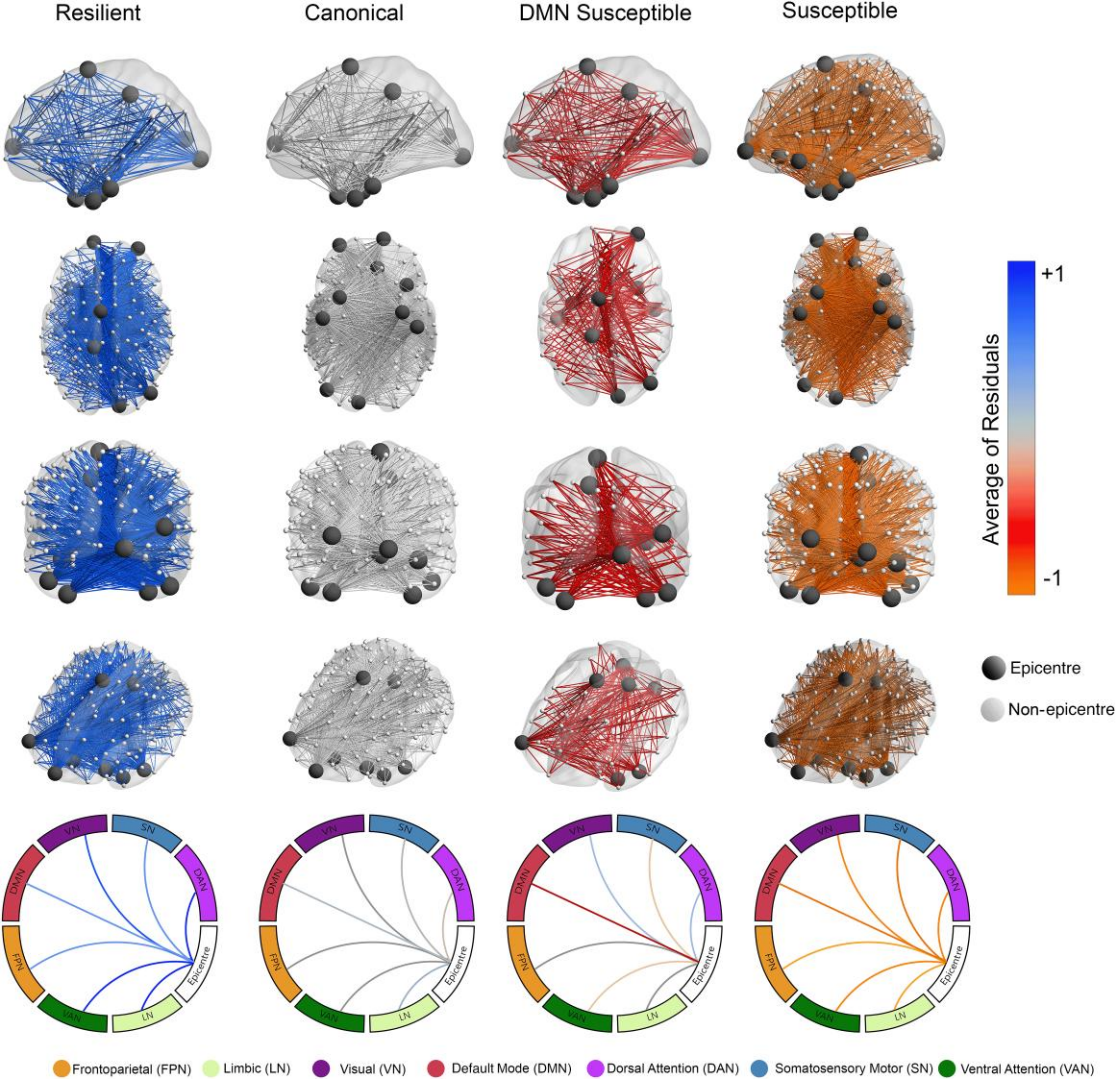
**Brain networks visualize tau spreading and functional disconnection relationships in Aβ-positive participants.** Compared to the canonical group, the resilient group has lower functional disconnection to epicentres given the level of tau spreading through functional connections (*n* = 94). The susceptible group has higher than expected functional disconnection compared to the canonical group given the tau spreading. The DMN-susceptible group has higher functional disconnection between tau-PET epicentres and non-epicentre regions in the DMN network given the relative tau spreading (linear regression). The scale represents mean tau spreading and functional disconnection relationship residuals in each of the seven networks (visual, somatomotor, dorsal attention, ventral attention, limbic, FPN and DMN). DMN, default mode network.

Herein, I characterize the four groups of tau spreading and functional disconnection relationship mismatch. The group close to the regression line in all networks with the smallest residuals is named canonical. The canonical group defined a condition where relative functional disconnection was statistically commensurate to the level of tau spreading. The other three groups were compared to the canonical group by tau spreading and functional disconnection relationship residuals in functional networks across all participants (Fig. 5), visualizing connections to epicentres where disruption is greater or less than what is observed in the canonical group given the tau spreading level.

There is one group with less functional disconnection relative to their tau spreading level compared to the canonical group (positive residuals), thus classified as resilient to tau spreading. Two groups had worse functional disconnection than typical for their level of tau spreading (negative residuals) and were considered susceptible to tau spreading. The group that had worse disruption FC to all networks given the level of tau spreading named the susceptible group. The other group had worse FC between epicentres and non-epicentre regions within the DMN network and thus named the DMN-susceptible group.

### Cluster groups have differences in Aβ but not tau pathology

**Figure 6:**
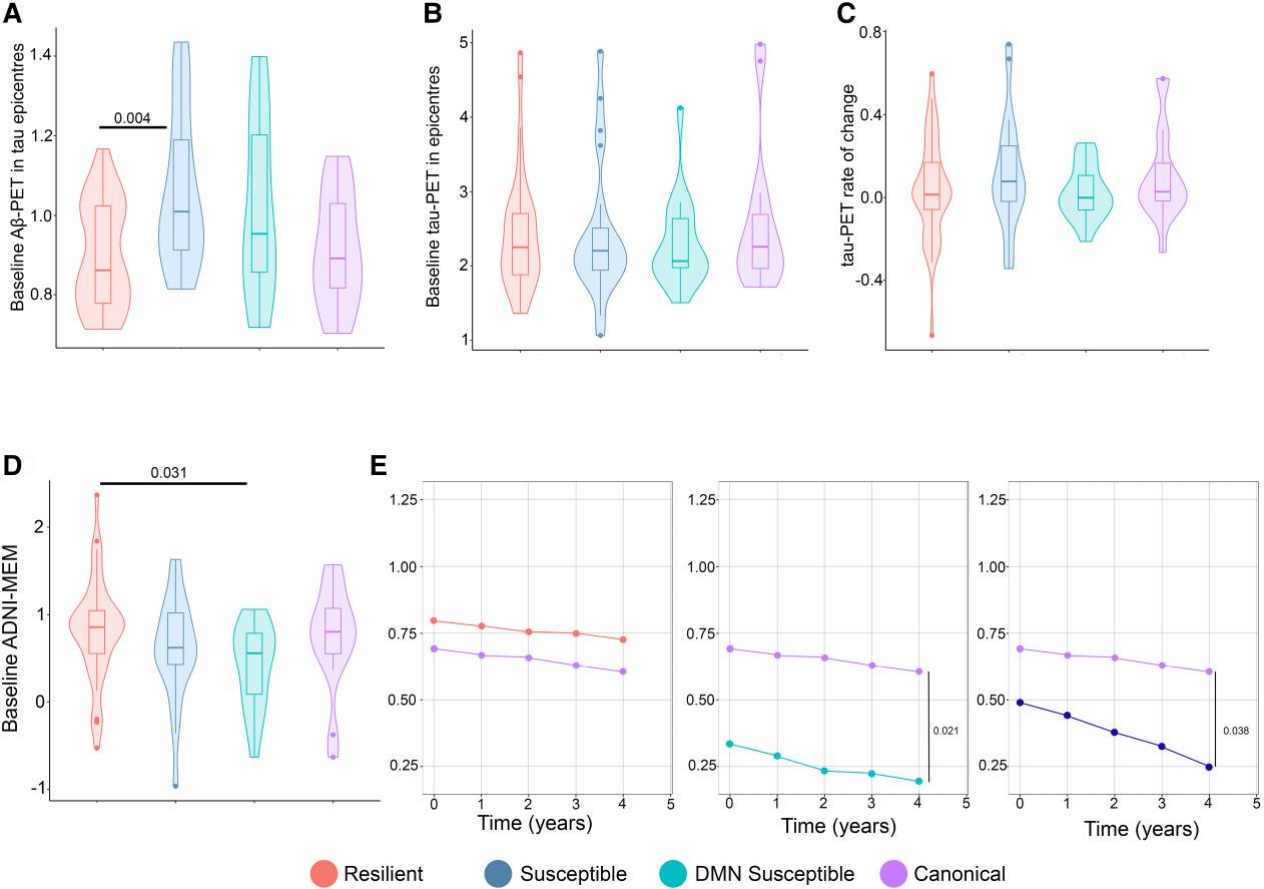
**Alzheimer’s disease pathological hallmarks and cognitive decline based on tau spreading and functional disconnection relationship.** (**A**) Baseline Aβ-PET in tau epicentres graphed in Aβ-positive participants (ANOVA, *n* = 94). The resilient group had lower baseline Aβ-PET in tau epicentres compared to the susceptible groups (**A**). (**B** and **C**) Baseline tau-PET in epicentres and tau-PET rate of change graphed in Aβ-positive participants (ANOVA, *n* = 94). The clustered groups were similar in baseline tau-PET in epicentres and rate of change in Aβ-positive participants (**B** and **C**). (**D**) Baseline ADNI-MEM graphed in Aβ-positive participants (ANOVA, *n* = 94). The resilient group had a higher ADNI-MEM score compared to the susceptible group in Aβ-positive participants (**D**). (**E**) Longitudinal cognitive decline in the clustered groups graphed in Aβ-positive participants (linear mixed effects, *n* = 51). The susceptible groups had faster cognitive decline compared to the canonical group in Aβ-positive participants (**E**). Box plots show mean as the middle box line, first quartile (Q1) and third quartiles (Q3) as box edges (denoting the IQR) and whiskers as the minimum–maximum points and outliers based on thresholds < Q1 − 1.5 (IQR) or >Q3 + 1.5 (IQR). *P*-values of significant differences in pairwise comparisons between the clustered groups by two-tailed likelihood ratio tests after covariate and multiple test (Bonferroni) adjustment are shown. Covariates include age, sex, education and APOE ε4 allele. Aβ, amyloid-beta; DMN, default mode network.

I evaluated whether clustering in tau spreading and functional disconnection relationship residuals was driven by Aβ or tau pathology. There was no difference in baseline tau-PET and rate of change between the clustered groups in the Aβ-positive and Aβ-negative groups (Fig. 6B and C; Supplementary Fig. 5B and C). However, the susceptible group had higher Aβ-PET in tau epicentre compared to the resilient group in Aβ-positive participants (Fig. 6A). Same results were replicated in Aβ-negative individuals (Supplementary Fig. 5A).

Additionally, I investigated the cross-sectional and longitudinal cognitive decline among the clustered groups. The susceptible group had a lower ADNI–MEM score compared to the resilient group in Aβ-negative, and while the DMN-susceptible group had lower ADNI-MEM score compared to the resilient group in Aβ-positive participants (Fig. 6D; Supplementary Fig. 5D). Then, I compared longitudinal cognitive trajectories with linear mixed effects models. Compared to the canonical group, both the susceptible groups had a faster cognitive decline in Aβ-positive and all participants (Fig. 6E).

### Exploratory analysis of copathology factors on tau spreading and functional disconnection relationship

**Figure 7:**
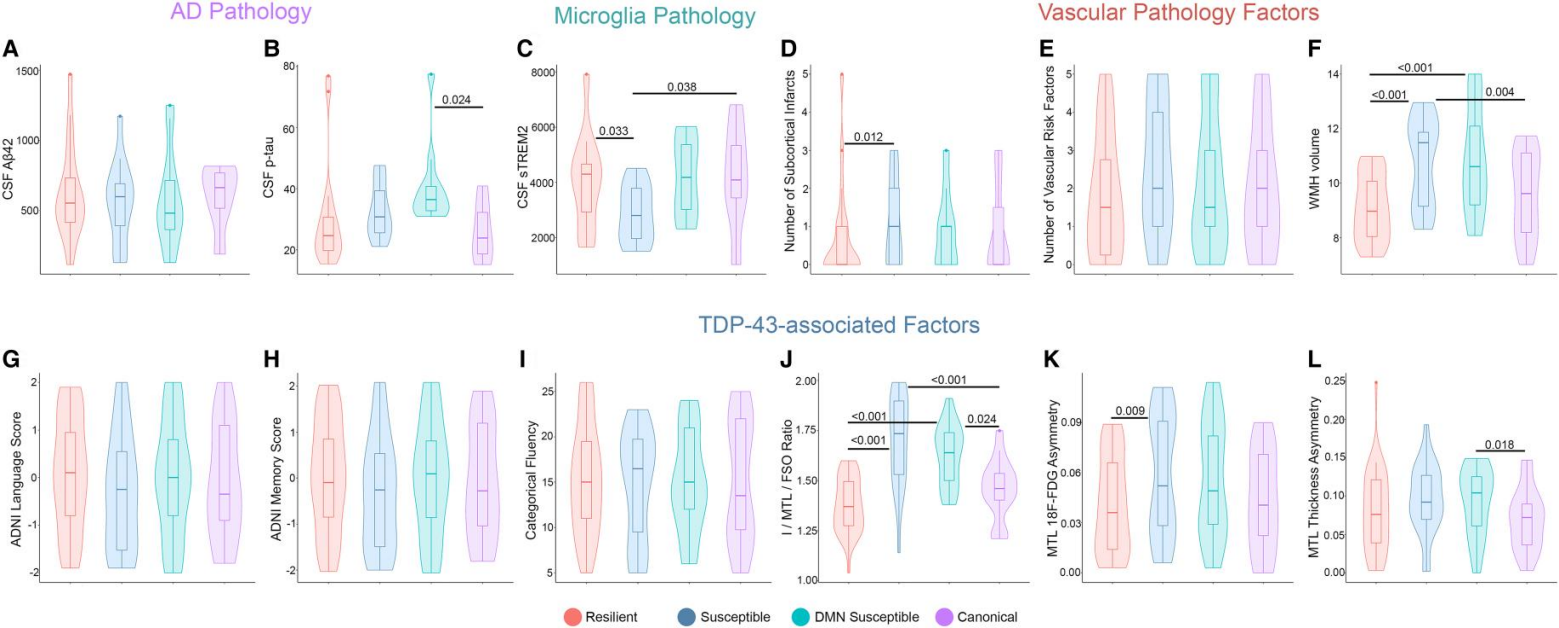
**Exploratory analysis of Alzheimer’s disease CSF biomarkers, microglia, vascular and TDP-43 copathologies based on tau spreading and functional disconnection relationship in Aβ-positive participants.** (**A** and **B**) Levels of CSF Aβ42 and p-tau are graphed (ANOVA, *n* = 74). The levels of CSF Aβ42 were similar among the clustered groups (**A**). The DMN-susceptible group had a higher level of CSF p-tau compared to the canonical group. (**C**) Level of sTREM2 as an indicator of TREM2-related microglia activity graphed among the clustered groups (ANOVA, *n* = 59). The susceptible group had lower CSF sTREM2 compared to the resilient and canonical groups. (**D–F**) Subcortical infarcts, vascular risk factors and WMH volume were compared based on tau spreading and functional disconnection relationship (ANOVA, *n* = 42). Susceptible subjects had a higher number of subcortical infarcts compared to the resilient group (**D**). The volume of WMH was higher in the susceptible groups compared to the resilient and canonical groups (**F**). Categorical fluency, ADNI language and memory scores were similar between the clustered groups (**G–I**; ANOVA, *n* = 66). Both the susceptible groups had larger I/MTL/FSO ^18^F-FDG ratios compared to the resilient and canonical groups (**J**; ANOVA, *n* = 53). Susceptible subjects had worse MTL ^18^F-FDG asymmetry compared to the resilient group (**K**; ANOVA, *n* = 53). Also, DMN-susceptible subjects had worse MTL thickness asymmetry compared to the canonical group (**L**; ANOVA, *n* = 67). Box plots show mean as the middle box line, first quartile (Q1) and third quartiles (Q3) as box edges (denoting the IQR) and whiskers as the minimum–maximum points and outliers based on thresholds < Q1 − 1.5 (IQR) or >Q3 + 1.5 (IQR). *P*-values of significant differences in pairwise comparisons between the clustered groups by two-tailed likelihood ratio tests after covariate and multiple test (Bonferroni) adjustment are shown. Covariates include age, sex, education, Aβ-PET and APOE ε4 allele. sTREM2, soluble triggering receptor expressed on myeloid cell 2; WMH, white matter hyperintensity; Aβ, amyloid-beta; DMN, default mode network; MTL, medial temporal lobe; FSO, frontal supraorbital; ADNI, Alzheimer’s Disease Neuroimaging Initiative.

Given that the susceptible groups had worse functional disconnection than expected given their tau spreading and faster cognitive decline, I hypothesize that other copathology might have a role in driving advanced functional disconnection. The level of CSF Aβ42 was similar between the cluster groups (Fig. 7A). However, Aβ-positive DMN-susceptible participants had higher CSF p-tau levels compared to the canonical group (Fig. 7B). I explored the frequency of APOE ε4 allele carriers and found that both the susceptible groups had a higher number of participants with APOE ε4 allele compared to the resilient and canonical groups (Supplementary Fig. 4A).

I investigated the level of CSF sTREM2 as an indicator of microglia pathology in the clustered groups. Aβ-positive-susceptible participants had a significantly lower level of CSF sTREM2 compared to the resilient and canonical groups (Fig. 7C). Also, the susceptible group had a higher number of subcortical infarcts compared to the resilient group in Aβ-positive participants (Fig. 7D). Moreover, there was a higher volume of white matter hyperintensity (WMH) in Aβ-positive-susceptible individuals compared to the resilient and canonical groups (Fig. 7E and F).

In the next step, I investigated how mixed proteinopathies may contribute to susceptibility to disruption in FC. While there was no definitive marker for proteinopathies such as TDP-43 in ADNI, I used several suggestive imaging and cognitive assessments to provide some indications for TDP-43 pathology.^35^ I explored the possibility of limbic-predominant age-related TDP-43 encephalopathy (LATE) copathology in the susceptible groups. I used the I/MTL/FSO ratio, defined as worse MTL and frontal supraorbital (FSO) hypometabolism relative to the inferior temporal gyrus (I). A higher I/MTL/FSO ratio indicates worse MTL hypometabolism and was shown to be correlated to LATE in previous studies.^36,37^ Both the Aβ-positive-susceptible groups had larger I/MTL/FSO ^18^F-FDG ratios compared to the resilient and canonical groups (Fig. 7J). Asymmetric hippocampal sclerosis is commonly present in LATE so I evaluated MTL asymmetry metrics for ^18^F-FDG hypometabolism and atrophy. Aβ-positive-susceptible participants had worse MTL ^18^F-FDG asymmetry compared to the resilient group (Fig. 7K). Also, the DMN-susceptible group had worse MTL thickness asymmetry compared to the canonical group among Aβ-positive participants (Fig. 7L). I further investigate memory phenotypes of LATE.^37^ However, there was no difference in categorical fluency, ADNI language and memory scores between the clustered groups (Fig. 7G–I).

## Discussion

The major aim of the present study was to explore the local effect of tau aggregate spreading on the FC between the epicentre and non-epicentre regions in Alzheimer’s disease. Using longitudinal molecular imaging and fMRI data, I first found that the spreading of tau aggregates through functional networks (assuming tau spread from epicentre to non-epicentre regions) is associated with reduced FC of non-epicentre to epicentre regions. Second, I found that Aβ in tau epicentre regions mediated the local effect of tau spreading on FC. Third, the clustering analysis exploring the mixed effect of other factors on FC revealed that the APOE ε4 allele, TREM2-related microglia activity, vascular risk factors and TDP-43 pathology were associated with susceptibility to brain functional disconnection. The present study provides a distinct view of how tau pathology might affect FC in Alzheimer’s disease progression. For the first time, I investigated the potential effect of tau spreading through functional networks on the involved connections only rather than networks not mediating tau spreading or having a little role. The present neuroimaging was conducted based on the spatial resolution of cerebral regions and prominent fibre pathways and was not aimed to comprehensively probe all possible cellular mechanisms or to quantify their effect. Instead, I designed the study based on the hypothesis that the spread of tau pathology across neurons contributes to the progression of Alzheimer’s disease while keeping in mind that other additional factors such as regional susceptibility, local spread and other underlying mechanisms may be involved in tau propagation. Another finding of the current study was the association between longitudinal changes in tau-PET accumulation and FC to epicentres, which is in line with the previous studies and suggests that functional architecture is a significant modifier of tau pathology.^9^

While it is believed that both Aβ and tau pathology might affect FC, there might be a more critical role for tau considering the fact that it is more strongly associated with cognitive decline and transneuronal spreading.^38-40^ A previous study demonstrated that lower FC in the DMN and the salience network, which are Aβ preferentially deposition areas, is associated with higher tau accumulation in the inferior temporal cortex.^41^ Moreover, the result of this study revealed that the association between tau and FC might be modulated by the Aβ pathology. Furthermore, the findings from a recent task-based fMRI study in individuals without cognitive impairment and with mixed Aβ status suggest that the tau accumulation in the memory system of the MTL is linked to increased FC in both the MTL and the posteromedial hubs of the DMN.^42^ It is worth noting that the initial accumulation of both tau and Aβ, despite occurring in different brain regions, seems to be negatively correlated with cognitive function in the preclinical stage of Alzheimer’s disease. Additionally, results from another study involving older and younger adults without cognitive impairment demonstrated that tau pathology plays a pivotal role in disconnecting the hippocampus from other regions of the MTL during healthy aging, resulting in impaired memory performance.^43^ The inability of pathological tau to stabilize microtubules and resulting impaired axonal transport can further increase synaptic loss, neurodegeneration and impairment of cognition.^39,44,45^ Although my study showed that tau spreading from epicentre to non-epicentre is specifically related to disconnection between these regions regardless of Aβ status, the association was partially mediated by Aβ in individuals with pathological levels of Aβ. However, given the 11% mediation effect, there is a need for further study to confirm the significant pathophysiological mediation role for Aβ. As such, tau spreading might affect FC, which can be facilitated by Aβ in early-stage Alzheimer’s disease. This may accord with activity-dependent accumulation of tau and Aβ where hyperactivity as a result of Aβ in the early stage Alzheimer’s disease might be involved in faster spreading of tau across networks and consequently more damage to the functional connections.^34,46-48^ Furthermore, it is hypothesized that Aβ might remotely and locally increase tau spreading and further accelerate functional disconnection.^49^ Together, the current study provides a better view of the spatial effect of tau pathology and the mediating role of Aβ on FC and then cognitive impairment.

Relative to the canonical group, Aβ-positive DMN-susceptible individuals had higher CSF p-tau and faster cognitive decline. It strengthens the notion that soluble p-tau concentration might lead to more functional disconnection due to transsynaptic tau spreading. In the initial stages, the accumulation of p-tau seeds associated with Aβ is taken up by neurons, initiating the misfolding and aggregation of tau within a specific brain region, and then, a higher soluble p-tau results in an accelerated local aggregation and spreading of tau.^25^ Also, in preclinical Alzheimer’s disease, higher levels of CSF p-tau and lower levels of Aβ_42_ were independently associated with disrupted FC in the anterior and posterior (PCC) hubs of the DMN and medial temporal areas.^50,51^ While my findings suggest that Aβ might be related to susceptibility to functional disconnection, but they do not specify the DMN susceptibility to disrupted connectivity. However, increased soluble p-tau in the early stage of Alzheimer’s disease taken up by neurons might lead to accelerated misfolded tau aggregation and more vulnerability to synaptic dysfunction.

I probed the potential effect of several other copathologies including vascular diseases, microglia activity and TDP-43 on the link between tau spreading and FC to tau epicentres. While all participants and Aβ-negative individuals did not differ in clinical vascular risk factors and microglia activity, Aβ-positive-susceptible participants showed higher CSF sTREM2 as an indicator of specific domains of microglia activity, subcortical infarcts and WMH volume. Furthermore, susceptible participants among all analysed groups demonstrated higher TDP-43 pathology markers. These findings suggest that non-Alzheimer’s disease copathologies might be a leading factor towards more severe disruption in FC related to tau spreading. Other common copathologies might be driving factors for resilience or vulnerability to functional disconnections such as abnormal microglia activity and vascular disease, which are common in Alzheimer’s disease. This view is supported by previous studies showing that clinical vascular risk factors and TREM2 deficiency can disrupt FC.^52-54^ Furthermore, I found that the susceptible group had CSF p-tau compared to the canonical group, which suggests that a higher soluble form of tau pathology can lead to more neurodegeneration given the expected tau pathology. In combination with the findings of a previous study by Pichet Binette *et al*.,^25^ which showed that higher soluble tau can further mediate tau spreading across functional connections, therapeutic interventions acting on clearance and inhibition releasing soluble form of tau aggregates might be beneficial.

My analysis of the resilient and susceptible groups supports the continued investigation of genetic and epigenetic features that influence the relationship between cell-to-cell tau spreading and FC. Note that both the susceptible groups tended towards a higher frequency of APOE ε4 allele carriers in individuals with or without pathologic levels of Aβ. It has been described that the APOE ε4 allele affects brain connectivity in known early-stage Alzheimer’s disease regions.^55^ Moreover, a different gene expression in various brain regions is associated with shred susceptibility to Alzheimer’s disease pathology such as tau deposition and neurodegeneration.^56-58^ The mechanistic pathway of vulnerability to disruption in FC in Alzheimer’s disease progression encompasses many different factors such as genetic features, copathologies, neuroinflammation and Alzheimer’s disease core pathology hallmarks, which warrant further research.

My study faced several limitations. First, I focused on FC, which is largely, but not completely, consistent with difussion tensor imaging (DTI)-measured structural connectivity.^59^ This may be partly determined by the technical limitation of DTI in detecting crossing fibres or short-range cortico-cortical connections.^60^ Second, on another hand, fMRI assesses the temporal association between regions with multisynaptic rather than direct connection.^61^ Moreover, regions with low measurable tau burden mainly subcortical regions were removed altogether from my model. Also, the study was designed based on the hypothesis that transneuronal tau spreading solely contributes to Alzheimer’s disease progression while other additional factors such as regional susceptibility, local spread and other underlying mechanisms may be involved in tau pathology development and further disruption in FC. Furthermore, the current study used cross-sectional fMRI data, which limited the determination of cause–effect relationships. Also, it should be noted that APOE ε4 allele carriers exhibit different PET findings compared to APOE ε4 allele non-carriers.^62^ Meanwhile, the main strength of the present study is the integration of cross-sectional and longitudinal imaging data to investigate the direct and local effects of connectivity-mediated tau spreading on the involved functional connections only. This gives us a unique view of how cell-to-cell propagation of tau pathology might affect synaptic function. Also, clustering participants based on the relationship between tau spreading and functional disconnection may have a practical value for biomedical research, particularly by allowing clinical trials to quantify heterogeneity across the spectrum of Alzheimer’s disease. For example, the susceptible groups may exhibit mixed pathologies along with tau pathology, leading to a reduced study power and complicating measuring the effect of experimental treatments targeting single pathways.

In conclusion, reconciling both Aβ statuses, I proposed an integrative model of how tau spreads through connections, FC, Aβ fibrils, genetic and other mixed pathologies interrelated. My findings provide strong support for the notion that tau spreading through connection is locally associated with disrupted FC between tau epicentre and non-epicentre regions independent of Aβ pathology. Also, I defined several groups based on the relationship between tau spreading and functional disconnection, which provides a quantitative assessment to investigate susceptibility or resilience to functional disconnection related to tau spreading. I showed that Aβ, other copathologies and the APOE ε4 allele can be a leading factor towards vulnerability to tau relative functional disconnection. Intervention limiting the tau spreading mechanism through functional networks^46^ or decreasing amyloid-induced hyperactivity, which may accelerate tau spreading, could be a potential candidate.^48,63^ Also, my findings could be a starting point for precision medicine that targets other potential factors leading to more vulnerability to functional disconnection and cognitive decline.

## Supplementary Material

fcae198_Supplementary_Data

## Data Availability

The data sets generated and/or analysed during the current study are available in the ADNI repository, https://adni.loni.usc.edu/. The data sets used and/or analysed during the current study are available from the corresponding author upon reasonable request. The codes used in this study are available via https://github.com/fardinnabizadeh/Disruption-in-functional-networks-mediated-tau-spreading-.
