# Supplementary material for: Disruption in functional networks mediated tau spreading in Alzheimer’s disease
Source: Brain Commun. 2024 Jun 13;6(4):fcae198. doi: 10.1093/braincomms/fcae198 (PMC11227975; doi:10.1093/braincomms/fcae198)
Supplement: fcae198_Supplementary_Data [file fcae198_supplementary_data.pdf]

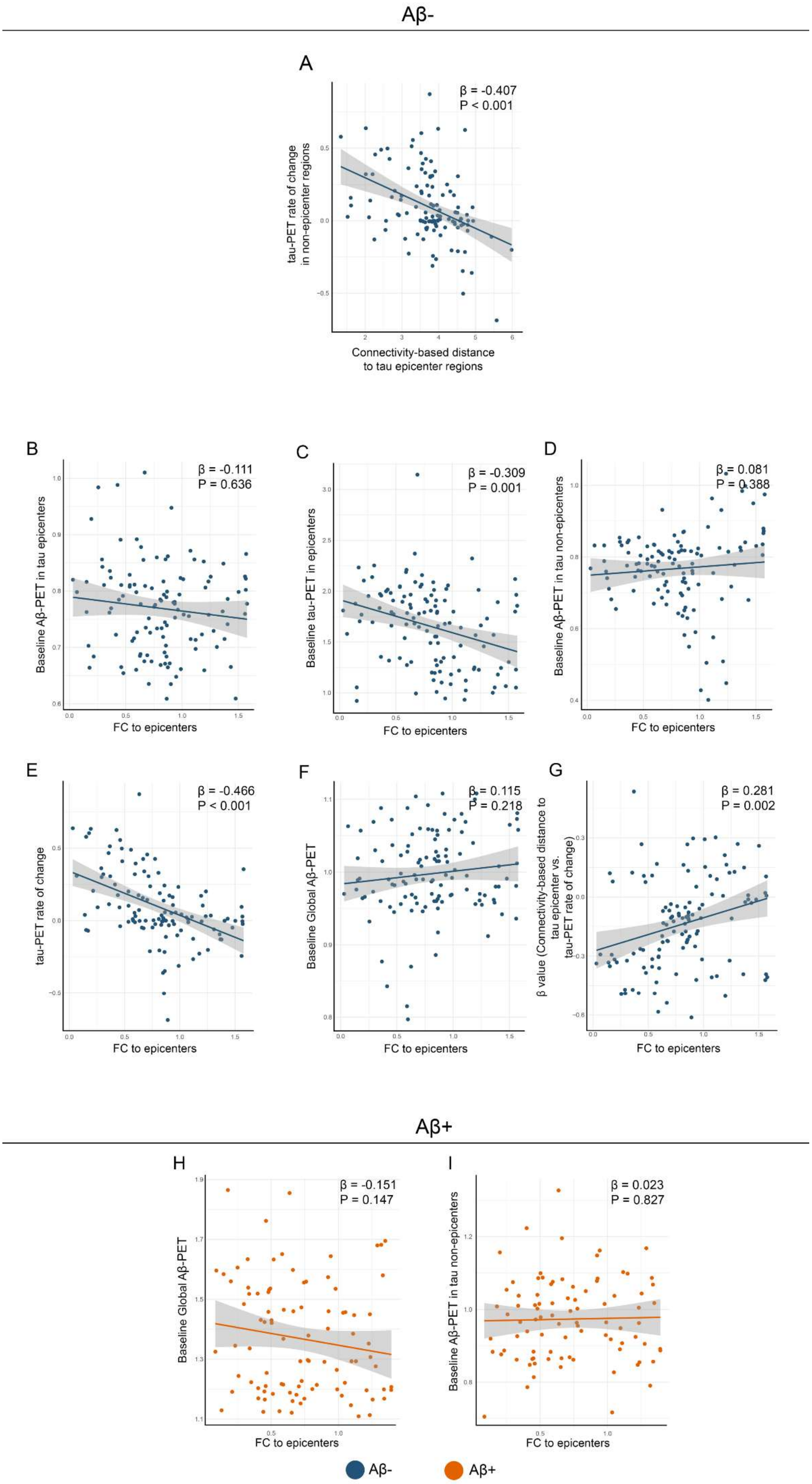

Aβ-
A
β = -0.407
P < 0.001
tau-PET rate of change in non-epicenter regions
Connectivity-based distance to tau epicenter regions
B
β = -0.111
P = 0.636
Baseline Aβ-PET in tau epicenters
FC to epicenters
C
β = -0.309
P = 0.001
Baseline tau-PET in epicenters
FC to epicenters
D
β = 0.081
P = 0.388
Baseline Aβ-PET in tau non-epicenters
FC to epicenters
E
β = -0.466
P < 0.001
tau-PET rate of change
FC to epicenters
F
β = 0.115
P = 0.218
Baseline Global Aβ-PET
FC to epicenters
G
β = 0.281
P = 0.002
β value (Connectivity-based distance to tau epicenter vs. tau-PET rate of change)
FC to epicenters
Aβ+
H
β = -0.151
P = 0.147
Baseline Global Aβ-PET
FC to epicenters
I
β = 0.023
P = 0.827
Baseline Aβ-PET in tau non-epicenters
FC to epicenters
Aβ-
Aβ+

Supplementary figure 1. Functional connectivity to epicenters is associated with AD pathological hallmarks. (A) The group-level analysis of the correlation between connectivity-based distance to the tau epicenters in Aβ-negative participants (Linear regression, n = 117). **(B-I)** Association between AD pathological hallmarks and FC to epicenter is shown on the scatter plot (Linear regression, n = 117). All linear regressions performed were two-sided, without adjustment for multiple comparisons, and error bands correspond to the 95% confidence interval. The linear models adjusted for the effect of age, sex, education, and APOE ε4. Aβ beta-amyloid, PET positron emission tomography, fMRI functional Magnetic Resonance Imaging, FC functional connectivity.

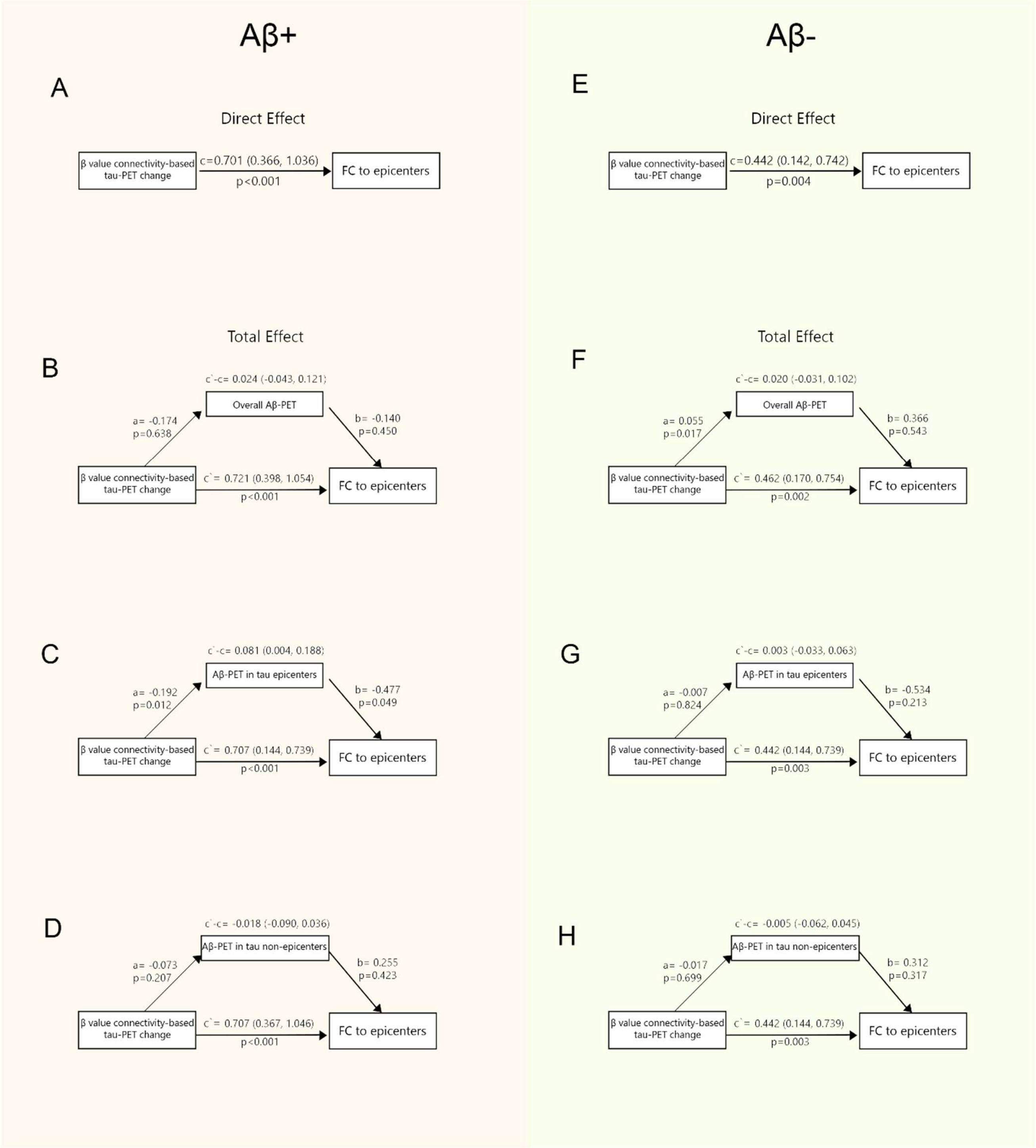

Supplementary figure 2. Aβ in tau epicenters mediates the association between tau spreading and functional connectivity to epicenters. (A and E) The direct effect of β value connectivity-based tau-PET change and FC to epicenters are depicted (Aβ-positive n = 94, Aβ-negative n = 117). **(B-D and F-H)** Analyses are shown with Aβ as mediator. **(C)** Aβ in tau epicenters mediated the relationship between β value connectivity-based tau-PET change and FC to epicenters in Aβ-

positive participants. The mediated effect is designated c-c'. The remaining effect of β value connectivity-based tau-PET change on FC to epicenters after adjusting for the mediator is designated c′. 95% confidence intervals derived from 1000 simulations are reported in parentheses. The direct effect of β value connectivity-based tau-PET change on the mediator is a, and the direct effect of the mediator on FC to epicenters is b. Aβ beta-amyloid, PET positron emission tomography, FC functional connectivity.

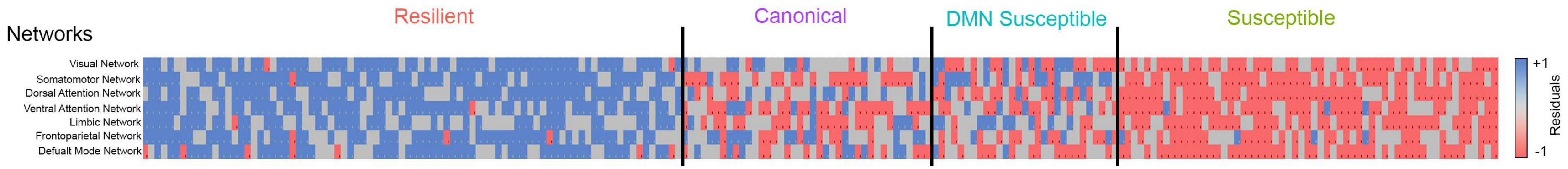

Supplementary figure 3. Analysis of tau spreading and functional disconnection relationship residuals and clustering. Heatmap of network residuals of tau spreading and functional disconnection relationship across clusters and 7 networks (Visual, somatomotor, dorsal attention, ventral attention, limbic, frontoparietal, and default mode network) (n = 211).

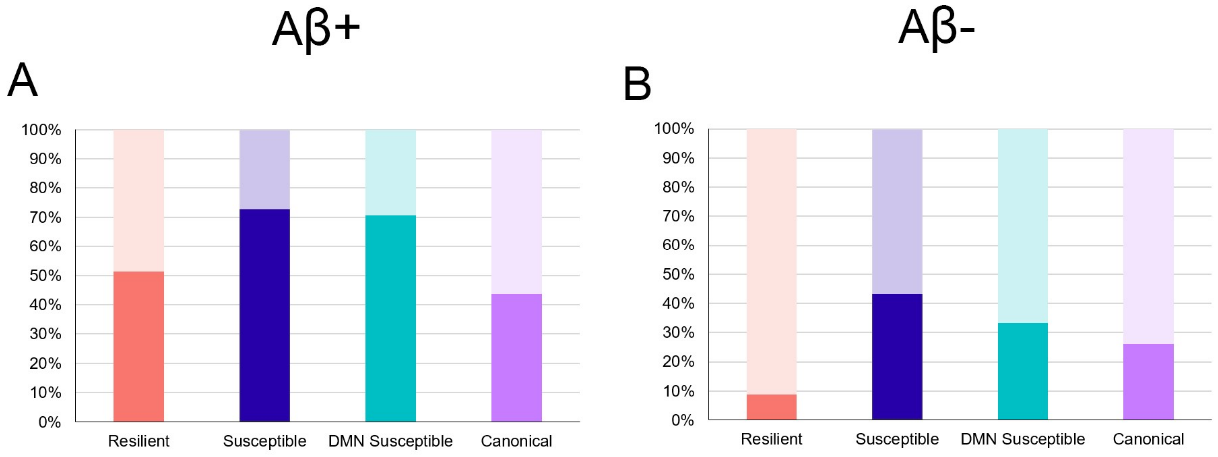

Supplementary figure 4. Frequency of APOE ε4 in clustered groups. (A-B). Both susceptible groups had higher number of subjects with APOE ε4 allele compared to resilient and canonical groups ($p<0.001$) ($\chi2$, Aβ-positive n = 94, Aβ-negative n = 117).

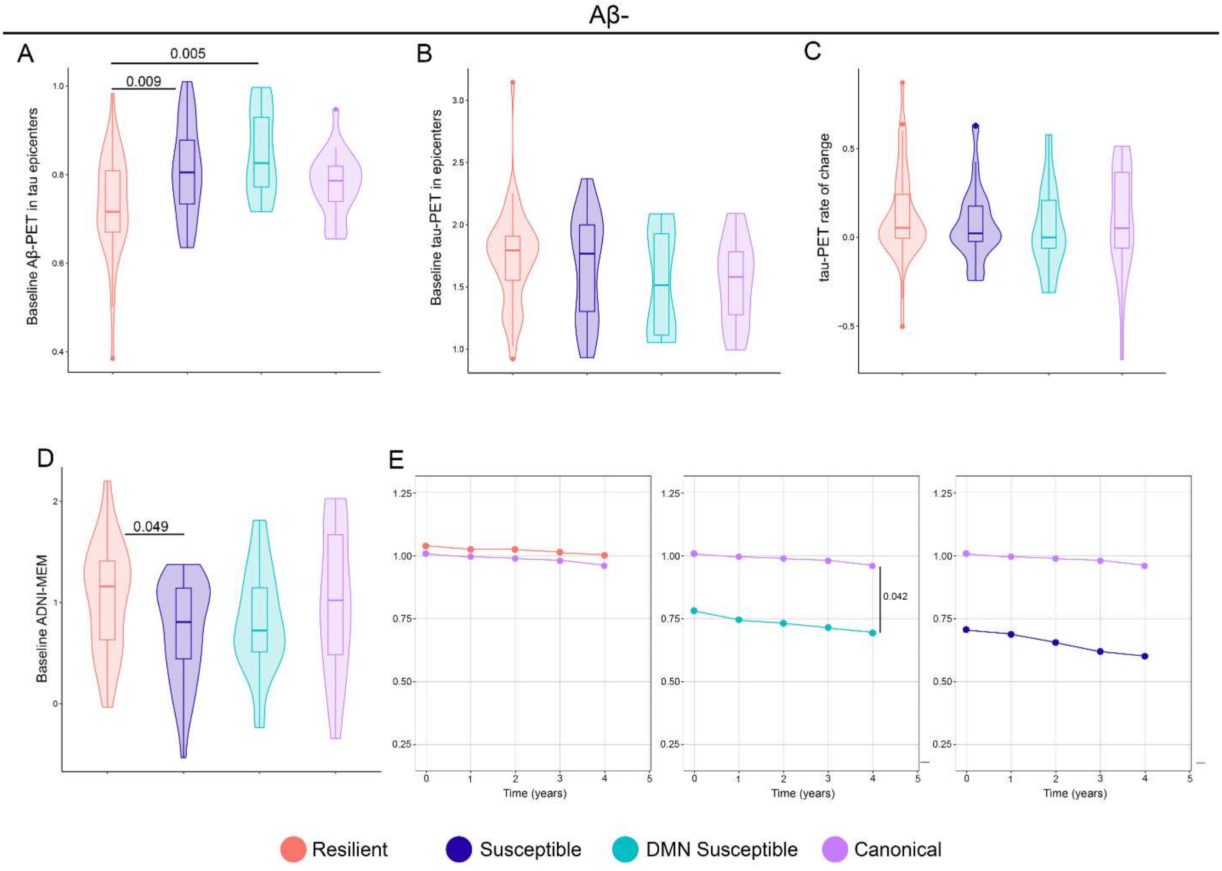

Supplementary figure 5. AD pathological hallmarks and cognitive decline based on tau spreading and functional disconnection relationship. (A) Baseline Aβ-PET in tau epicenters graphed in Aβ-negative participants (ANOVA, n = 117). (**B and C)** Baseline tau-PET in epicenters and tau-PET rate of change graphed in Aβ-negative participants (ANOVA, n = 117). **(D)** Baseline ADNI-MEM graphed in Aβ-negative participants (ANOVA, n = 117)**. (E)** Longitudinal cognitive decline in clustered groups graphed in Aβ-negative participants (linear mixed-effects, n = 78). Box plots show mean as the middle box line, first quartile (Q1) and third quartiles (Q3) as box edges (denoting the interquartile range, IQR), whiskers as the minimum/maximum points and outliers based on thresholds <Q1 − 1.5(IQR) or >Q3+ 1.5(IQR). P-values of significant differences in pairwise comparisons between clustered groups by two-tailed likelihood ratio tests after covariate and multiple test (Bonferroni) adjustment are shown. Covariates include age, sex, education, and APOE ε4. Aβ beta-amyloid, PET positron emission tomography, DMN default mode network.
